# Social media reduction and friendship outcomes in youth: the roles of resilience and usage motives

**DOI:** 10.3389/fpsyg.2026.1962459

**Published:** 2026-10-13

**Authors:** Karina Schowalter, Patrik Reiter, Lisa Klümper, Maria Agthe

**Affiliations:** 1 UMIT TIROL—Private University for Health Sciences and Health Technology, Institute for Psychology, Hall in Tirol, Austria; 2 Department of Psychology, University of Wuppertal, Wuppertal, Nordrhein-Westfalen, Germany

**Keywords:** communication, friendship quality, friendship quantity, psychological resilience, social media reduction, social media usage motives, youth

## Abstract

Evolved affiliation motives drive people to seek belonging and monitor social inclusion. Social media (SM) can support these motives by facilitating contact but may also undermine offline friendships. We tested whether friendship quality/quantity changed after a two-week SM reduction and whether SM-use motives and psychological resilience moderated changes. Children (10+), adolescents, and young adults were recruited in schools and universities. At T1, an experimental-group (*n* = 480) and a control-group (*n* = 293) completed online or paper-and-pencil surveys; the experimental-group was asked to abstain from SM for 2 weeks, while controls continued using. At T2, 287 experimental- and 196 control-participants completed the survey. Participants who used SM reported motives in an open-ended item at T2. We examined behavior-based reductions (i.e., actual voluntary reduction), allowing us to speak to real-world change but does not support causal inference from assignment. Variance analyses showed significant time and reduction-group interactions for both outcomes: moderate reductions (reduced use ≥ 30 min/day) were associated with small improvements in friendship quantity and quality, whereas stricter limits (use < 30 min/day) yielded no benefit. At baseline, resilience was positively linked to friendship quantity and quality but did not predict or moderate T1–T2 change. Among continued users, T2 friendship quality (controlling T1) was positively associated with communication motives and negatively with selective restriction, while quantity showed no motive links. Overall, resilience appears to be a general resource for friendship quality and may buffer motive–relationship links. Understanding youths’ SM motives may promote healthier use and guide ethical platforms and education design, especially for vulnerable youth.

## 1 Introduction

Friendship involves not only number of friends but who they are and the perceived quality of interactions (Hartup, 1996). High-quality friendships, characterized by prosocial behavior and intimacy (Berndt, 2002), support social and psychological development across all ages (Parker and Asher, 1993; Hartup, 1996; Berndt, 2002). Evolutionarily, friendship reflects reciprocal support grounded in social exchange/credit (Tooby and Cosmides, 1996).

Friendship quality is closely associated with well-being. Healthy peer relationships associate with greater subjective well-being, happiness, and self-esteem in adolescents, although longitudinal evidences remain limited (Alsarrani et al., 2022). Existing longitudinal findings suggest that low perceived social acceptance and poor dyadic friendship quality in early adolescence predict later social anxiety, whereas high late-adolescent friendship quality predicts greater job satisfaction, future well-being, and lower romantic insecurity as young adults (Shah et al., 2024). Thus, adolescent friendship quality represents an important target for prevention/intervention.

Given SM’s risks and benefits (Bekalu et al., 2019), examining its role in adolescents’ lives is crucial for protecting friendship quality. Male adolescents report lower self-esteem, well-being, and friendship closeness on days with more SM use (van der Wal et al., 2025), reducing mental health. Adolescents report mainly negative SM effects on friendships and mental health, though some report benefits and adverse effects; these differences may depend on SM usage motives.

SM platforms utilize evolved affiliation motives by providing tools for social monitoring, self-presentation, reciprocal communication and arousal regulation (avoiding boredom). These platforms allow users to maintain large social networks, and may mimic ancestral environments where monitoring group dynamics was essential for survival. However, the often superficial nature of online interactions may dysregulate these mechanisms, leading to maladaptive outcomes such as fear of missing out (FoMO) or excessive social comparison (Tufan et al., 2025; Rajguru et al., 2026). Understanding how these underlying evolutionary motives interact with individual differences, like resilience, and exploring how SM usage motives reflect these fundamental motives, is critical for designing interventions that promote adaptive SM-use.

Bekalu et al. (2019) identified motives for SM use, including staying connected/informed, shaping identity/status through self-presentation/social enhancement, maintaining relationships (compensating for limited offline-contact), and regulating emotions (e.g., stress, loneliness, low mood). Consequences of SM use partly depend on use-patterns: routine use tends to relate to better well-being, whereas strong emotional attachment relates to poorer outcomes. Similarly, Rajguru et al. (2026) identified motives associated with nomophobia (the fear of not having access to a mobile phone), including seeking belonging and validation, compensating for unmet offline social needs, maintaining connections over distance, avoiding face-to-face interactions (especially with social anxiety/low self-esteem), FoMO, and sensation-seeking/curiosity. Smartphones thus meet social, coping, and evolutionary needs for extraverts and introverts. Their findings suggest that unmet belonging increases phone-attachment, whereas resilience, supportive offline relationships, parental involvement/supervision and physical proximity to close others are protective (Rajguru et al., 2026). Together, these studies indicate that evolutionary, social and coping-related motives shape SM/smartphone-use, while the extent to which social needs are met offline may influence whether use remains functional or becomes problematic (Bekalu et al., 2019; Rajguru et al., 2026).

Methodologically, Mostyn Sullivan et al. (2025) developed the *Motives for Smartphone Use Questionnaire* (MSUQ) to explain young adults’ smartphone use, identifying seven factors: *Coping, Pass Time, Social, Social Comfort, Safety, Social Obligation*, and *Information Seeking*. Showing, Social Comfort and Social Obligation as distinct motives, previously undercaptured; Coping and Social Comfort were strongest predictors of problematic use. These studies show the need for nuanced SM research.

Overall, the implications of SM use for young people’s relationships may depend on usage motives and individual protective factors. The present study examines how usage reduction and specific usage motives relate to young peoples’ friendship characteristics, focusing on resilience as a protective factor that may promote friendship quality and quantity, subjective well-being, and mental health while attenuating detrimental effects of adverse patterns of SM use.

We examine cross-sectional associations between resilience and friendship outcomes at baseline, changes in friendship quantity and quality over time, and differential changes associated with SM reduction. We further test whether baseline resilience predicts or moderates changes in friendships. Finally, for participants using SM during the two-week period, we investigate whether SM use motives and resilience at T2 relate to friendship outcomes at T2, controlling for baseline-levels. Our focus is on how voluntary (rather than assigned) reduction relates to change, we interpret behavior-based reductions as observational.

## 2 Methods

### 2.1 Participants

Of the *N* = 773 participants who completed T1, *N* = 483 were reliably matched to T2 via personal codes and included; incomplete cases (i.e., participants without matched T1–T2 data) were excluded from the current analyses.

Non-inclusion mainly reflected late/non-completion of the questionnaire, absence at one wave (e.g., illness), or non-matchable personal codes due to typing errors (particularly at T1), so that comparisons between T1 and T2 were not possible. Very few were excluded for response issues (e.g., insults in open-ended fields, highly repetitive responding), and few were excluded during data collection for disruptive behavior in the study setting.

*N* = 483 participants categorized into three adapted adolescent age groups and one adulthood group (Salmela-Aro, 2011) can be seen in Table 1. Age was analyzed as continuous.

**Table 1:** Age and gender of the study participants.

Age group	*N*	Female	Male	Diverse	Age	*SD*	Range (years)
		*n* (%)	*n* (%)	*n* (%)	*M* (years)		
Early-adolescence	144	74 (51)	69 (48)	1 (1)	12.0	0.95	10–13
Middle-adolescence	164	85 (52)	77 (47)	2 (1)	14.9	0.80	14–16
Late-adolescence	112	63 (56)	47 (42)	2 (2)	17.7	0.72	17–19
Adulthood	63	50 (80)	12 (19)	1 (2)	23.2	4.16	20–46
Total	483	272 (56)	205 (42)	6 (1)	15.8	3.92	10–46

Middle school and high school students were recruited and surveyed at their respective schools, whilst university students were invited to take part in the study online.

The questionnaire was pre-tested to ensure understandability to the youngest participants (aged 10–11) resulting in minor linguistic changes of all scales. The study was approved by the Research Committee for Scientific Ethical Questions (RCSEQ, 3552/25) at the Private University for Health Sciences and Health Technology—UMIT TIROL.

### 2.2 Design and procedure

After completing the first questionnaire at T1, the experimental group were asked to voluntarily (and preferably completely) refrain from using SM, both active and passive consumption (such as watching someone else’s screens), for the next 2 weeks. The control group was asked to continue using SM as usual. After 2 weeks (T2), both groups were asked to complete the questionnaire again.

Both questionnaires included the following sociodemographic variables: participants’ age, biological sex, number of siblings, number of individuals residing in one’s household (not counting the participant) and the cohabitants relationship category (e.g., family, friends, colleagues, roommates, other), residential area (e.g., urban, suburban, rural, other), parent’s home country, and self-rated proficiency in German on a scale from one to five.

SM use was assessed by a German adaptation of the *social networking sites usage & needs scale* (SNSUN; Ali et al., 2020), maladaptive SM use was assessed using the *Social Media Use Disorder Scale for Adolescents* (SOMEDIS-A; Paschke et al., 2021). Next, participants were asked about their bed- and wake-up -time, as well as how recovered they felt after their night’s sleep on a regular weekday. If they felt unrecovered, they were asked if this was due to health problems. Depressive symptoms were screened using the German version of the *PHQ-9* (Patient Health Questionnaire-9; Löwe et al., 2003), self-esteem through an adaptation of the *Rosenberg-Scale* (RSES, von Collani and Herzberg, 2003) as well as two items of the *contingencies-of-self-worth-subscale* ‘Approval from Others’: “I don’t care if other people have a negative opinion about me. “and “I can’t respect myself if others don’t respect me. “(CSWS; Crocker et al., 2003), administered in German (Schütz and Sellin, 2003). General health and emotion regulation were assessed with the German adaptation of the *Emotion Regulation Questionnaire for Children and Adolescents* (ERQ-CA; Gullone and Taffe, 2012), emotional affect with the German version of *PANAS* (Breyer and Bluemke, 2016), stress with the German adaptation of the *Perceived Stress Scale* (PSS-10; Schneider et al., 2020) using a six-point Likert-scale, instead of a five-point Likert-scale (to avoid a tendency towards the middle). Resilience was assessed using the German short version *RS-11* (Schumacher et al., 2005). Friendship was measured by a self-developed scale (see 3.4). The second questionnaire (T2) included one additional question asking why SM had been used (if used). All items were framed for the past 2 weeks.

### 2.3 Intervention

Before T1, the experimental group received a school-based presentation clarifying which platforms counted as SM and which remained permitted during the two-week intervention. Online participants at universities (ages 18 and older) received written instructions at the beginning.

The intervention between the survey at T1 and T2 lasted 2 weeks. The platforms participants should abstain from using involved sharing and consuming text-posts, videos, and images (e.g., Facebook, Instagram, X, Snapchat, Pinterest, etc.). They were allowed to use communication platforms (e.g., WhatsApp) to stay in touch with family, peers, and schools but were asked to avoid groups or channels sharing media. The study also permitted using streaming services (e.g., Spotify, Netflix, etc.), restricting on YouTube—participants should completely avoid watching Shorts/Reels (or as much as possible). The study ensured participants could continue hobbies like gaming and allowed them to play online- or videogames but asked to avoid consuming content shared in chats (e.g., via Steam) resembling SM.

The overall goal of the intervention was avoiding long, mostly mindless consume of SM content and the phenomenon of “doom scrolling” (excessive consumption of negative online-news; Yasan-Ak et al., 2025), as much as possible, without sacrificing the ability to maintain contact with others or pursue hobbies.

### 2.4 Variables and scale development

Dependent variables were assessed with a 12-item friendship scale. We examined its structure at both T1 and T2 using exploratory factor analysis (EFA; PAF, promax rotation). Sampling adequacy was excellent at both time points [T1: KMO = 0.94; Bartlett’s *χ*^2^(66) = 4,588, *p* < 0.001; T2: KMO = 0.95; Bartlett’s *χ*^2^(66) = 5,188, *p* < 0.001]. The EFA suggested a two-factor, oblique solution reflecting Friendship Quantity and Friendship Quality. According to the Kaiser–Guttman criterion (eigenvalue > 1), two factors were retained as well. Items loaded on the same factors at both T1 and T2, indicating a stable structure. However, three items did not meet the simple-structure criterion (difference between primary and secondary loadings < 0.20) and were excluded. After evaluating scale reliability and item redundancy, we removed two additional items due to redundancy. The final solution (see Table 2) comprised two correlated subscales capturing Friendship Quantity (3 items) and Friendship Quality (4 items) with acceptable reliability.

**Table 2:** Descriptives and reliability of friendship quantity, friendship quality and resilience for both measurements (T1 and T2).

Scale	Item description	M T1	SD T1	Cronbachs *α* T1 (if item deleted)	*M* T2	*SD* T2	Cronbachs *α* T2 (if item deleted)	Original item
Quantity of friendship		3.50	1.20	0.799	3.58	1.22	0.809	
	I have many friends with whom I like to spend time with.			(0.697)			(0.736)	Ich habe viele Freunde, mit denen ich gerne Zeit verbringe.
	I have a big circle of friends.			(0.707)			(0.740)	Ich habe einen großen Freundeskreis.
	I have as many friends as I would like (to have).			(0.773)			(0.742)	Ich habe so viele Freunde, wie ich mir wünsche.
Quality of friendship		4.15	0.98	0.873	4.14	0.99	0.889	
	My friends like me as I am.			(0.856)			(0.879)	Meine Freunde mögen mich so, wie ich bin.
	I have friends who help me when I have problems.			(0.819)			(0.836)	Ich habe Freunde, die mir helfen, wenn ich Probleme habe.
	I have at least one friend whom I can completely confide in.			(0.872)			(0.876)	Ich habe mindestens einen Freund oder eine Freundin, der ich alles anvertrauen kann.
	Whenever I am sad or stressed, I have friends who are there for me.			(0.800)			(0.836)	Wenn ich traurig oder gestresst bin, habe ich Freunde, die für mich da sind.
Resilience		5.44	0.93	0.882	5.48	1.00	0.907	

Cronbach’s *α*, reliabilities and means in this table relate to T1. *N* = 483. The response format is a 6-point Likert-scale. English item wordings are provided for clarity only; the original German version was administered and used for factor-analytic validation.

### 2.5 Formation of usage motives

The motives for SM use were rated by three independent evaluators (two women, one man) on a scale of 0 = not present or 1 = present. They evaluated responses to an open-ended question: “Tell us why and for how long you have used SM over the past two weeks [e.g., out of boredom or because you wanted to distract yourself, etc.]. You can list multiple reasons.” The raters identified twelve categories (see Table 3).

**Table 3:** Interrater-reliability of usage motives.

Usage motive	Frequency	Interrater agreement	% overall agreement
	*N*	Fleiss’ *κ*	
Fun/to reduce boredom	275	0.77	83
Communication with others	125	0.86	92
Watching videos	57	0.72	91
Habitual use	23	0.62	93
Fear of missing out	31	0.59	93
School- or work-related use	29	0.75	96
Gaming	11	0.83	99
To pass the time while waiting	20	0.66	95
Sleep aid/insomnia	7	0.68	98
Restricted use of certain platforms = selective restriction	48	0.46	85
Maintaining visibility	14	0.68	97
Generating ideas for a creative hobby	16	0.72	97

### 2.6 Formation and use of social media reduction groups

Although participants were assigned to conditions at T1, only a subset in the reduction condition actually reduced their use. Because our focal construct is actual voluntary change in usage, we base our primary analyses on behavior-based reduction groups. We explicitly acknowledge that behavior-based comparisons are observational and can be influenced by self-selection; therefore, we refrain from causal language regarding intervention effects of assignment. Accordingly, we created a behavior-based measure of reduction that distinguishes three categories: (1) no reduction of SM use, (2) reduced use relative to T1 (daily use at least 30 min less than at T1), and (3) usage reduced to less than 30 min per day. This allows us to address the following questions: (1) Is there an actual reduction in SM use, depending on its extent, associated with the outcome variables (quantity/quality of friendships) at T2? (2) Do any significant correlations persist when relevant covariates (e.g., resilience, age, sex) are controlled for? (3) Do these correlations vary depending on specific motives for SM use?

## 3 Results

We report results by behavior-based reduction groups, reflecting our focus on the effects of voluntary reduction. These between-group differences are observational (i.e., not randomized contrasts) and are therefore not interpreted as causal intervention effects of assignment.

### 3.1 Differential changes in the quality and quantity of friendships over time

To examine whether quantity and quality of friendships changed from T1 to T2 depending on the SM reduction, we conducted paired *t*-tests within the three reduction-groups (no reduction, reduction relative to T1: ≥30 min/day less, reduced to <30 min/day; difference defined as T1 − T2).

Multivariate baseline-adjusted analyses showed both T2 friendship outcomes as dependents and both T1 friendship measures as covariates, the multivariate effect of reduction-group was significant [Pillai’s trace = 0.034, *F*(4, 956) = 4.12, *p* = 0.003]. *Post-hoc* univariate analyses of variance indicated significant reduction-group differences at T2 for both quantity, *F*(2, 478) = 5.89, *p* = 0.003, and quality, *F*(2, 478) = 6.62, *p* = 0.001, after controlling for baseline.

To unpack the time × reduction-group interactions, we examined within-group changes (paired tests; T1 − T2; seen in Table 4). In the relatively reduced group (≥30 min/day less), friendship quantity increased, *t*(165) = −3.63, *p* < 0.001, *d* = −0.28, and friendship quality increased, *t*(165) = −2.67, *p* = 0.004, *d* = −0.21. In the < 30 min/day group and in the non-reduction group neither quantity nor quality changed significantly (*p* > 0.05). Overall, the results suggest that relative SM reduction is associated with improvements in friendship quantity and quality, whereas strict reduction to <30 min/day shows no improvements.

**Table 4:** Results of the *t*-tests for different reduction groups and measurements (friendship QUAN and QUAL) over time (T1–T2).

Group	*n*	Method T1–T2	*t*(df)	*p*	*M*_diff_	95% CI	*d*
Non-reduced SM use	250	QUAN	0.35(249)	0.638	0.017	[−0.102, 0.146]	0.02
		QUAL	2.15(249)	0.984	0.100	[0.011, 0.260]	0.14
Relatively reduced SM use	166	QUAN	−3.63(165)	**<0.001**	−0.201	[−0.437, −0.126]	−0.28
		QUAL	−2.67(165)	**0.004**	−0.136	[−0.316, −0.053]	−0.21
SM use reduced to less than 30 min per day	67	QUAN	−0.75(66)	0.228	−0.104	[−0.333, 0.149]	−0.09
		QUAL	−0.03(66)	0.489	−0.004	[−0.243, 0.236]	−0.00

Significant results are printed in bold type. QUAL, quality of friendship; QUAN, quantity of friendship. 95% CI of Cohen’s *d*. Measure of effect size = Cohen’s *d*.

### 3.2 Correlation between resilience and friendship

To investigate whether resilience at T1 is related to friendship quantity or quality at T1, we examined the relationship in a generalized linear multivariate model with resilience (T1) as the predictor and both measures of friendship at T1 as dependent variables. The overall test was significant, Pillai’s *V* = 0.288, *F*(2, 480) = 97.10, *p* < 0.001. Univariate *post-hoc* tests revealed significant positive associations with both friendship quantity (T1), *F*(1, 481) = 118.00, *p* < 0.001, *η_p_*^2^ = 0.197, as well as with friendship quality (T1), *F*(1, 481) = 169.00, *p* < 0.001, *η_p_*^2^ = 0.260. Thus, resilience at T1 is substantially associated with higher friendship quantity and quality at T1.

### 3.3 The role of resilience for possible changes in friendship

To examine whether resilience at T1 was associated with changes in the quantity or quality of friendships between T1 and T2, we estimated regression-based change models in which T2 was controlled for T1.

For quantity, we predicted FQuantityT2 from FQuantityT1, Resilience (T1), and their interaction. The model explained 59.3% of the variance (*R^2^* = 0.593, *adj. R^2^* = 0.591). The overall model was significant, *F*(3, 479) = 233.00, *p* < 0.001. FQuantityT1 was positively associated with FQuantityT2 [*b* = 0.543, SE = 0.142, 95% CI (0.263, 0.822), *t*(479) = 3.82, *p* < 0.001], whereas neither the main effect of resilience [*b* = −0.146, SE = 0.097, 95% CI (−0.336, 0.045), *t*(479) = −1.50, *p* = 0.133] nor the FQuantityT1 × Resilience interaction [*b* = 0.045, *SE* = 0.027, 95% CI (−0.007, 0.097), *t*(479) = 1.71, *p* = 0.088] were significant.

For quality, we regressed FQualityT2 on FQualityT1 and Resilience (T1) and their interaction. The model explained 47.7% of the variance (*R^2^* = 0.477, *adj. R^2^* = 0.481). The overall model was significant, *F*(3, 479) = 148.00, *p* < 0.001. FQualityT1 strongly predicted FQualityT2 [*b* = 0.453, SE = 0.130, 95% CI (0.198, 0.708), *t*(480) = 3.49, *p* < 0.001], whereas neither the main effect of resilience [*b* = −0.151, SE = 0.106, 95% CI (−0.360, 0.058), *t*(479) = −1.42, *p* = 0.157] nor the FQualityT1 × Resilience interaction [*b* = 0.045, SE = 0.026, 95% CI (−0.003, 0.098), *t*(479) = 1.86, *p* = 0.064] were significant. In summary, we found no evidence that Resilience at T1 predicts or moderates the changes in the quantity or quality of friendships from T1 to T2 (controlling for baseline levels). Durbin–Watson values near 2 indicate no autocorrelation between residuals for both regressions.

### 3.4 Usage motives and resilience as predictors for friendship

To examine whether SM usage motives at T2 are associated with friendship quantity or quality at T2, controlling for the baseline (T1), we estimated a linear regression model for each variable. We restricted these analyses to participants who did not reduce their SM use. The following motive analyses are exploratory, with small cell sizes and many predictors entered simultaneously.

To predict the quantity of friendships at T2 (dependent variable), we estimated a linear regression model with our 12 dichotomous usage motives (0/1; see Table 3), resilience (T2), the motive, sociodemographic covariates (sex, age, number of siblings), and the friendship quantity at T1 as a control variable. The overall model was significant, *F*(18, 229) = 24.70, *p* < 0.001, *R^2^* = 0.660, *adj. R^2^* = 0.634 (*N* = 248). Stability over time was pronounced: a higher quantity of friends at T1 predicted a higher quantity at T2, *b* = 0.784, SE = 0.045, 95% CI [0.696, 0.872], *t*(229) = 17.51, *p* < 0.001. Resilience (T2) showed a small positive additional association, *b* = 0.106, SE = 0.054, 95% CI [0.0001, 0.212], *t*(229) = 1.97, *p* = 0.050. None of the 12 usage motives was associated with the quantity at T2 (all *p* ≥ 0.11); similarly, sex, age, and number of siblings showed no effects. Assumptions of validity were met (multicollinearity: VIFs ≤ 1.12; independent residuals: Durbin-Watson = 2.04, *p* = 0.714).

For friendship quality at T2 (dependent variable), we used the analogous specification with quality at T1 as a control. The model was significant as well, *F*(18, 229) = 16.30, *p* < 0.001, *R^2^* = 0.561, *adj. R^2^* = 0.526. In addition to the clear stability from T1 to T2, *b* = 0.667, SE = 0.0568, 95% CI [0.555, 0.779], *t*(229) = 11.75, *p* < 0.001, resilience (T2) showed a positive additional association, *b* = 0.221, SE = 0.0488, 95% CI [0.125, 0.317], *t*(229) = 4.53, *p* < 0.001. Two motives were associated with quality at T2 independently of other predictors: “Communication with others” (positive), *b* = 0.223, SE = 0.102, 95% CI [0.022, 0.423], *t*(229) = 2.19, *p* = 0.030, and “selective restriction” (negative), *b* = −0.661, SE = 0.229, 95% CI [−1.112, −0.211], *t*(229) = −2.89, *p* = 0.004. Other motives, as well as sex, age, and number of siblings, were not significant (all *p* ≥ 0.09). Assumptions of validity were met (VIFs ≤ 1.16; Durbin-Watson = 1.94, *p* = 0.630).

The results suggest that friendship measures at T2 are primarily explained by T1 levels, controlling for baseline. Resilience at T2 adds slightly to quantity and positively to quality. Among usage motives, “Communication with others” is positively associated with friendship quality at T2, while “selective restriction” is negatively associated; no motives were additionally associated with quantity.

## 4 Discussion

Moderate, self-determined SM reductions (≥30 min/day less use) over 2 weeks were associated with small, significant gains in friendship quantity and quality; aligning with evidence that brief SM cuts reduce loneliness and depressive symptoms (Hunt et al., 2018; Lambert et al., 2022; Faulhaber et al., 2023). These benefits may reflect a sensible readjustment of evolved social monitoring mechanisms, as reducing passive scrolling and superficial interactions likely allowed participants to focus on higher-quality, face-to-face interactions, aligning with the important evolutionary function of friendships in fostering emotional intimacy and reciprocal support (Tooby and Cosmides, 1996).

By contrast, limiting use to <30 min/day did not improve friendship outcomes, suggesting highly restrictive limits may offer no benefit or even be counterproductive. Such restrictions may reduce awareness of and engagement with friends’ lives, weakening perceived closeness. Participants attempting to abstain entirely may have experienced stress from high self-restrictions when aiming at not using SM at all, and stress-related emotions and cognitions might have offset beneficial effects of SM reduction. Moderate, self-determined reductions may be less stressful while creating more opportunities for rewarding face-to-face interactions, which support mood and well-being (e.g., Forbes et al., 2023; Krach et al., 2010). Consistently, complete SM abstinence has been linked to increased negative affect and reduced life satisfaction (Vally and D’Souza, 2019). Although these effects were partly attributed to random assignment in prior studies, our design allowed participants to determine their reduction extent, reducing this concern.

The slight decline in friendship quality among participants who maintained their SM use may reflect reduced time for face-to-face interactions. Digital communications (Stieger et al., 2023) and SM use (e.g., Ai and von Mühlenen, 2025) may not provide the same positive effect as in-person contact and are linked to lower mood and loneliness (e.g., Riehm et al., 2019). Additional factors may include tensions between reducers and non-reducers (experimental group) and a one-week school break in controls may have contributed to the slight decline in friendship quality.

Among participants maintaining their SM use, friendship quality at T2 was positively associated with communication and negatively with selective restriction, consistent with evidence that sharing personal experiences fosters liking and trust (e.g., Hirschi et al., 2023). By contrast, restrictions are linked to unpleasant experience and lower mood, which might temporarily affect friendship perception. Friendship quantity at T2 was primarily predicted by T1, indicating relatively stable networks over 2 weeks.

### 4.1 The role of resilience regarding SM use and friendship

Resilience, as an evolved trait, plays a critical role in maintaining social bonds. Its positive association with friendship quality and quantity at T1 suggests that resilient youth is better equipped for navigating social challenges. This aligns with research linking adolescent friendship quality and resilience (e.g., van Harmelen et al., 2021), and resilience to emotion regulation in adolescents (Surzykiewicz et al., 2022) and positive emotion use during recovery from negative experiences (Tugade and Fredrickson, 2004). Resilience may buffer the negative effects of maladaptive usage patterns, such as FoMO or selective restriction, by promoting adaptive coping strategies and emotional stability. These findings highlight resilience as a protective factor in both offline and online contexts, supporting the evolutionarily advantageous maintenance of high-quality social bonds.

Despite these cross-sectional links (de Terte et al., 2014), resilience did not predict changes in friendship quality/quantity between T1 and T2, suggesting resilience contributes to baseline differences in friendship quality/quantity rather than short-term change. Thus, resilience may contribute to participant’s initial friendship levels but is not an independent predictor of change in this study.

### 4.2 Social media usage motives and friendship

Outcomes in SM reduction depend not only on the extent of reduction but also on usage motives, consistent with prior research (see Bekalu et al., 2019). Differential effects of SM usage motives on friendship quality highlight the importance of how social media aligns/misaligns with evolved social needs. Motives such as “Communication” may enhance friendship quality by facilitating reciprocal exchanges, consistent with the evolutionary function of social bonds (Hirschi et al., 2023). In contrast, motives like “selective restriction” may disrupt social monitoring processes, leading to perceived failures in maintaining connections. These findings emphasize the need for interventions that address the interplay between evolutionary motives and SM usage patterns. Research on phubbing/SPA suggests that FoMO fosters problematic use, undermining friendship, while contextual factors like being phubbed moderate these links (Tufan et al., 2025). This supports integrating motive- and behavior-level constructs in future models.

At T2, friendship quantity was driven primarily by T1 levels, indicating stable networks over 2 weeks. By contrast, friendship quality related to specific motives: “Communication” was positively associated with T2 quality, aligning with findings that SM facilitates contact and communication (e.g., Reece et al., 2023), whereas “selective restriction” was negatively associated. One explanation is compensatory shifts to other platforms, yielding a perceived failure to meet intended goals.

Other motives showed no significant links to friendship outcomes, potentially due to small subgroup sizes (limited statistical power) or heterogeneous effects not captured by dichotomous measures. For instance, “maintaining visibility” may depend on social comparison: rating one’s own posts as worse than others may elicit negative affect, whereas favorable self-perceptions may have positive consequences.

### 4.3 Limitations and future research

Moderate SM reduction was associated with improvements in friendship characteristics. The amount and motives of SM use, and individual resources like resilience, appeared more relevant to friendship quality than quantity. The complex role of usage motives needs further investigation.

Some limitations should be considered. All measures, including usage data, were self-reported, and may have been affected by recall bias and socially desirable responding, despite assurance of anonymity and requests for honest answers. Partial self-selection into non-reduction, and non-enforced reductions (to minimize reactance) may have introduced systematic differences (e.g., personality, self-regulation). Future work should identify characteristics facilitating voluntary reduction. Attrition from T1 to T2 and handling of missing data (e.g., excluding incomplete cases) may bias estimates; accordingly, we avoid strong causal conclusions. Lastly, analyses involving specific motives were exploratory, with small cells (reduced power/increased uncertainty) and multiple tests (greater false-positive risk); findings are interpreted cautiously.

Our study supports moderate, realistic reductions rather than rigid limits <30 min/day align with evolved social needs, particularly of youth. Interventions may be more effective when reducing adverse passive scrolling while preserving adaptive relationship-supportive communication, allowing individuals to prioritize meaningful, reciprocal interactions and social bonds over superficial online-connections. By fostering resilience and encouraging prosocial, communicative SM use, and by platform designs promoting active, relationship-focused communication over passive consumption, (particularly young) people’s navigation in digital environments should be better aligned with human’s evolved social needs.

## Data Availability

The datasets presented in this article are not readily available because personal codes in dataset, identifying children and adolescents under the age of 18. Data could be provided if needed after deleting the personal codes (identifying variable). Requests to access the datasets should be directed to Karina Schowalter, karina.schowalter@gmail.com.
